# Nature-based tourism as therapeutic landscape in a COVID era: autoethnographic learnings from a visitor’s experience in Iceland

**DOI:** 10.1007/s10708-022-10713-5

**Published:** 2022-07-27

**Authors:** Allison Williams, Rannveig Ólafsdóttir

**Affiliations:** 1 School of Earth, Environment & Society, McMaster University, Hamilton, Ontario Canada; 2 Department of Geography and Tourism Studies, Institute of Life and Environmental Sciences, University of Iceland, Reykjavik, Iceland

**Keywords:** Therapeutic landscapes, Autoethnography, Iceland, Sustainable nature-based tourism

## Abstract

One of the few silver linings in the COVID pandemic has been a new appreciation for, interest in, and engagement with nature. As countries open, and travel becomes accessible again, there is an opportunity to reimagine sustainable nature-based tourism from a therapeutic landscape lens. Framed within the therapeutic landscape concept, this paper provides an autoethnographic account of a visitor’s experience of three different natural landscapes in Iceland shortly after the country’s fourth wave of the pandemic. It adds to the understanding of the healing effects of the multi-colored natural landscapes of Iceland. The natural landscapes of interest herein include: the southern part of the Westfjörd peninsula, Jökulsárlón glacial lagoon, and the Central Highlands. In totality, the natural, built and symbolic environments worked in synchronicity to produce three thematic results: *restoration, awe* and *concern*, all which provided reduced stress, renewed attention, as well as enhanced physical and psycho-social benefits for the autoethnographic visiting researcher. Implications of these restorative outcomes for sustainable nature-based tourism in a post-COVID era are discussed. This paper highlights how health and tourism geographers can work collaboratively to recognize, protect, and sustain the therapeutic elements of natural landscapes, recognized as a cultural ecosystem service. In so doing, such collaborations can positively influence sustainable nature-based tourism development and consumption through proper and appropriate planning and development of such tourism destinations.

## Introduction

As challenging as COVID-19 has and, in many ways, continues to be for the world’s population, it has taught us a few things as well. For instance, we have learned that: work can take place anywhere, the benefits of technology and telemedicine are indisputable, self-care is not self-indulgent, and nature matters. With the challenges and hardships associated with stay-at-home orders, social isolation, and the stress of the pandemic itself, many have found a new appreciation for nature and are consequently more motivated to preserve it. By most measures, the planet did well during COVID, with limited air travel and consumption, movement of people, and limited interruption to wildlife habitat, for example (Gunton et al., 2021; AARP, 2021). The renewed interest in natural areas is likely to create a new boom in nature-based tourism worldwide. COVID has brought a time of pause for tourism, providing the time and attention to better improve the sustainable planning, development, and management of nature-based tourism in a post-COVID era.

In addition to the impacts caused by the pandemic, several other forces are at play in the quest to protect nature; these include respite from an increasingly urbanizing and technologically driven world, and worry about the loss of nature due to environmental and climate change. Nature provides an opportunity to slow down, offering a time-out from the demands of technology and city life. Buckley (2022) notes that research evidence indicates that both nature and adventure tourism contribute to positive mental health given that enjoyment promotes wellbeing. Climate change is another force at work. In addition to those motivated by, ‘last chance tourism’ (e.g. Abrahams & Hoogendoorn, 2021; Salim & Ravanel, 2020), tourists are increasingly seeking opportunities to breath fresh air in a world facing a growing number of wildfires every year (National Interagency Fire Centre, 2021). Seeking natural environments through nature-based tourism has been gradually rising in recent decades (e.g. Ólafsdóttir et al., 2021; Sæþórsdóttir, 2010) and is likely to rise more rapidly in the wake of the pandemic. This is especially so in Iceland, a country which has experienced an explosion in tourism since the late turn of the century (Icelandic Tourism Board, 2021). One of the primary reasons for the burst in Iceland’s tourism sector pre-COVID is due to its natural environment, and especially its pristine and wild character, which has long formed the backbone of the country’s tourism industry. This is reflected in the many marketing slogans for Icelandic tourism including: ‘all natural’, ‘unspoiled wilderness’, ‘pure nature’, and ‘Europe’s last wilderness’ (Sæþórsdóttir & Karlsdóttir, 2009; Jóhannesson et al., 2010; Ólafsdóttir et al., 2016).

As a concept, wilderness is, however, heavily disputed, particularly in relation to the uses and management of wilderness areas in the Anthropocene (Saarinen, 2016, 2019). Still, no universally accepted definition of the concept exists. Wild and untamed nature represents, nonetheless, an environment that is becoming increasingly rare in our industrialized world and, consequently, a precious environment which a growing number of tourists are seeking. The value of areas where it is possible to get in close contact with nature is increasing, and especially so in wilderness settings where it is possible to enjoy solitude and tranquillity. In this paper we will generally refer to natural areas given that our sites of concern have varying degrees of wild and untamed nature.

**Fig. 1:**
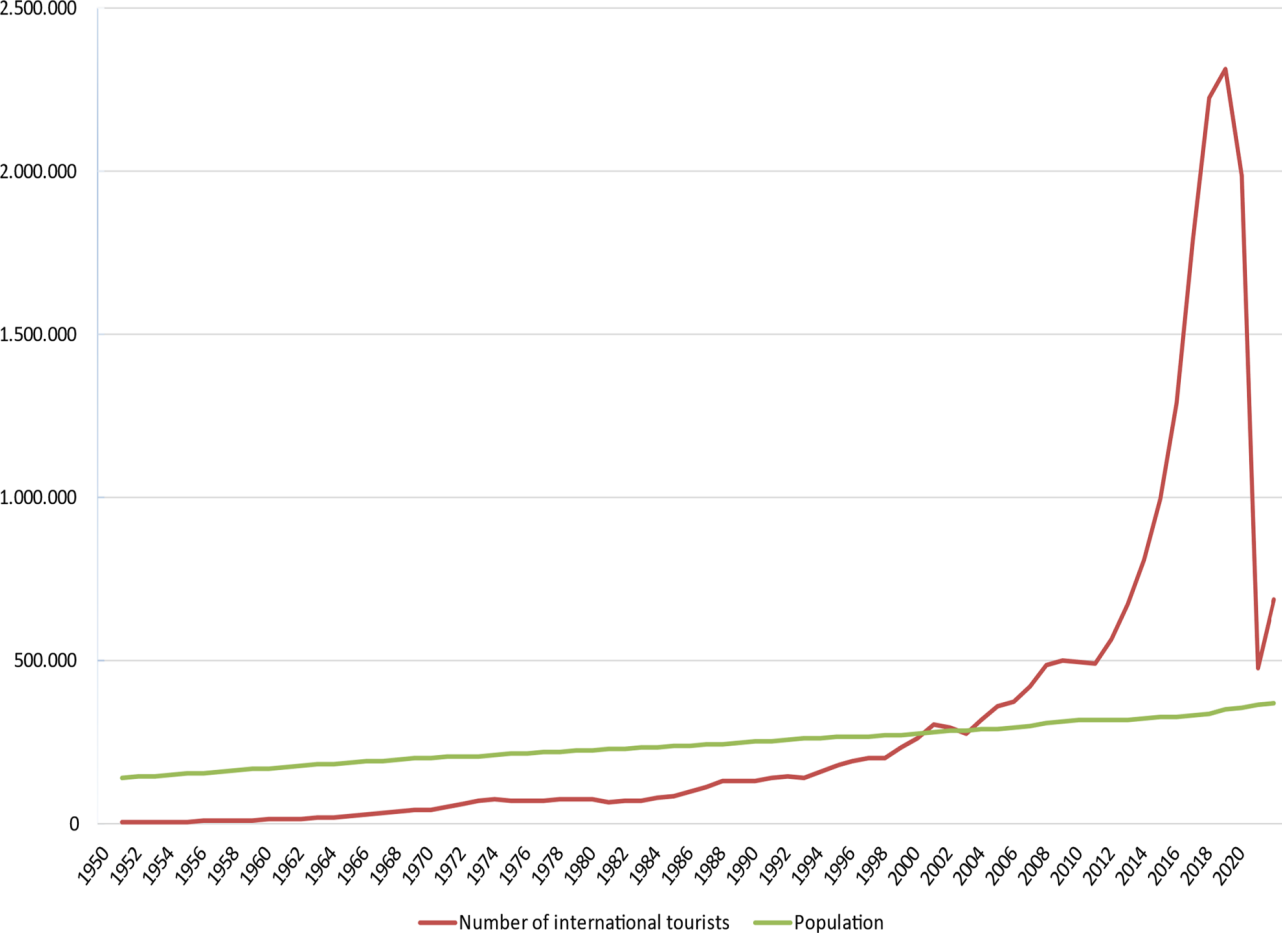
Numbers of Foreign international visitors to Iceland in relation to population development 1950–2021 (Data sources: ITB, 2022; Statistics Iceland, 2022)

**Fig. 2:**
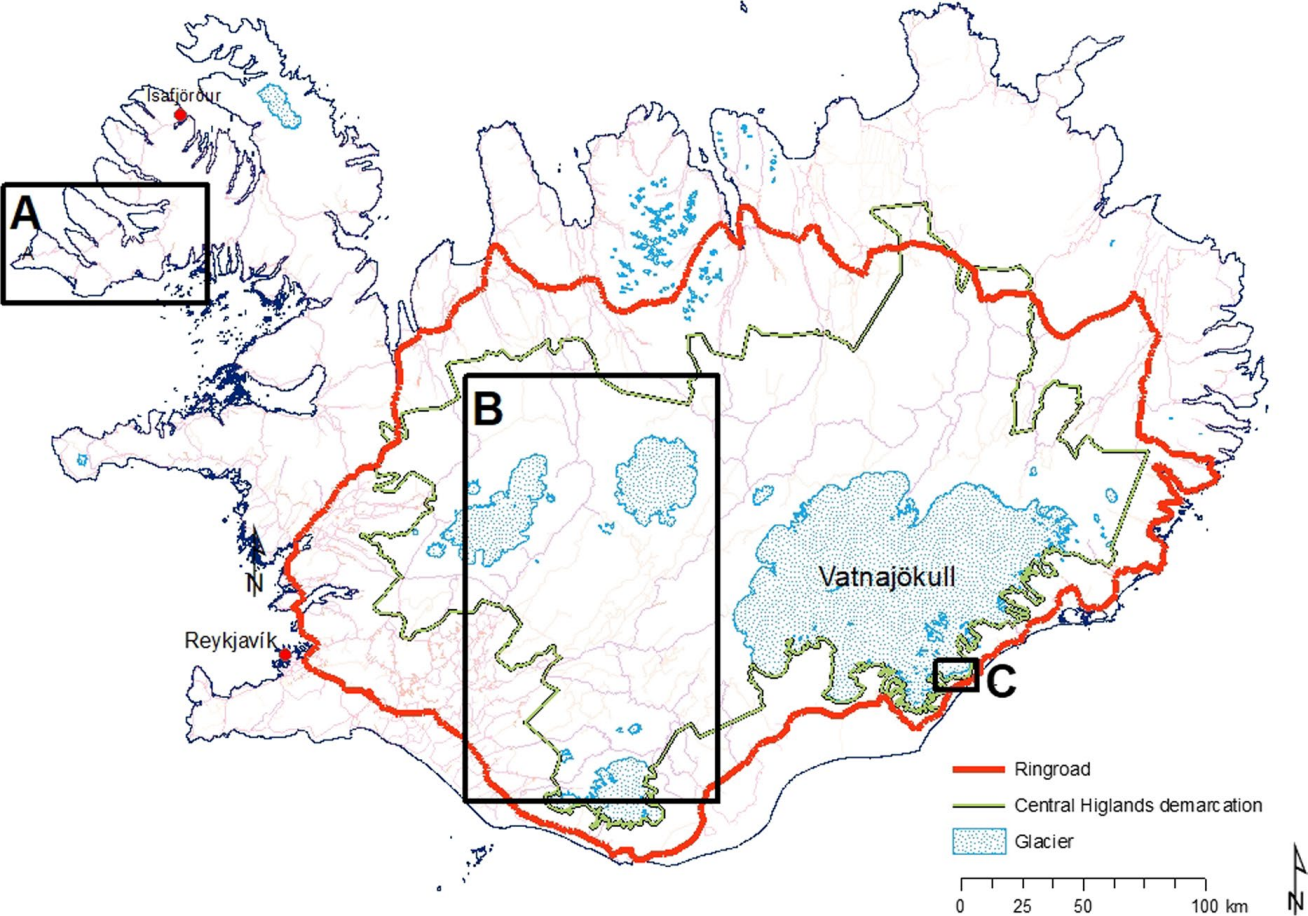
The location of the three study sites used in the study. **A** the southern part of the Westfjords; **B** part of the Central Highlands, and **C** Jökulsárlón glacial lagoon

Framed within the therapeutic landscape concept, this paper provides an autoethnographic account of a visitor’s experience of three different natural landscapes in Iceland shortly after the country’s fourth wave of the pandemic. This paper highlights how health (first author) and tourism (second author) geographers can work collaboratively to recognize, protect, and sustain the therapeutic elements of natural landscapes and, by so doing, inform sustainable nature-based tourism through illuminating the health benefits of natural landscapes. This is particularly important within the context of the growing nature-based tourism sector in the fragile Arctic and Sub-Arctic regions experiencing elevated climate change impacts. Iceland, located just south of the Arctic Circle, is a sparsely populated country with only 376,000 inhabitants (Statistics Iceland, 2022) sharing a land area of 103,000 km^2^. Throughout Icelandic history inhabitants have mainly been located along the coastline, leaving the interior highlands an uninhabited wilderness. Over the course of the past few decades, tourism has grown rapidly in Iceland; for example, 1950 had approximately 4,000 international visitors compared to nearly 2.4 million in 2018, which is sevenfold the country's population the same year. The number of international visitors dropped somewhat in 2019, and then collapsed in 2020 due to COVID (Fig. 1). In 2021 the number rose to nearly 700 thousand (Iceland Tourist Board, 2022). The escalating growth in tourism since the turn of the century triggered overtourism in some of Iceland's most popular destinations (Ólafsdóttir et al., 2021). There are many indications that tourism will grow rapidly again as travel restrictions due to the COVID epidemic are eased. Likewise, natural destinations like Iceland will be highly sought after following a long period of restraint. As countries open, and travel becomes accessible again, there is an opportunity to reimagine sustainable nature-based tourism from a therapeutic landscape lens. Furthermore, using an autoethnographic account of a visitor’s experience of three diverse natural landscapes in Iceland, the therapeutic benefits of these natural environments are better understood. The three natural sites selected to fulfil the aim of this study include: the southern part of the Westfjords peninsula, the Central Highlands, and the Jökulsárlón glacial lagoon (Fig. 2). Both authors travelled together to the Westfjords. The autoethnographic visiting researcher travelled to the Jökulsárlón glacial lagoon on her own and was accompanied by an Icelandic super jeep driver in the Central Highlands. The first author is the visiting health geographic autoethnographic researcher with knowledge about therapeutic landscape theory. As a tourism geographer and a native of Iceland, the second author provided important country context while situating the findings within the larger nature-based sustainable tourism literature.

A short review of the literature pertaining to therapeutic landscapes will provide the background for the paper. Next, the autobiographical approach will be described. Results are presented via three themes: *restoration, awe,* and *concern*. Implications for nature-based tourism in a post-COVID era are discussed. The methodology and results are written in first person, given that they were experienced by the first author, as a visiting researcher to Iceland.

## Therapeutic landscapes and nature affiliation

Recognized as significant contribution that health geographers have made to the broad study of health, therapeutic landscapes theory is a conceptual framework for the analysis of physical (natural and built), social and symbolic environments, as they contribute to physical and mental health and well-being in places (Bell et al., 2018; Gesler, 1992). Traditionally, applications have been realized in the following four areas: traditional landscapes (i.e., reputed for health and/or healing, such as shrines, pilgrimages, etc.), natural/pristine/wild areas, landscapes for the marginalized (i.e., mentally ill, autistic, etc.), and applications to health care sites (i.e., hospitals, long-term care facilities, etc.) (Williams, 1999, 2007; Bell et al., 2018). A 2018 scoping review of the therapeutic landscape literature noted that, as a health geographical concept, therapeutic landscapes: offer in-depth insight into experiential, embodied and emotional geographies; promote awareness of place as both therapeutic and exclusionary, and; continues to be a relevant and lively field of inquiry across health geography (Bell et al., 2018).

Certainly, natural environments have long been shown to be therapeutic landscapes (Williams, 2007). Natural pristine and wild places of geographical splendor may also be understood as an important cultural ecosystem service. Cultural ecosystem services provide a range of varied benefits to humans, such as recreational, tourism, aesthetic, and spiritual benefits, all which enhance human and physical wellbeing (Millennium Ecosystem Assessment, 2005). In totality, cultural ecosystem services intersect with therapeutic landscapes in the many benefits places have, all of which contribute to enhanced mental and physical wellbeing.

Within the ongoing evolution of therapeutic landscapes theory, recent focus has been the examination of the coloured elements of landscapes, or the palettes of place (Bell et al., 2018); the colours blue and green have been particularly highlighted as promoting health and well-being (Foley et al., 2019). Within the context of Iceland, the significance of white and black landscapes was discussed by Brooke and Williams (2021), who recognized that many other natural landscapes of various colours have yet to be fully explored with respect to their therapeutic value, such as blue, green, yellow, brown, and grey. Azevedo (2020) recognizes wilderness as being exemplified by a range of colours, including white, blue, green and gold. We introduce a range of colours here within the context of health-enabling places.

## Methodology

To explore the beneficial impact of Icelandic landscapes in relation to tourism, an authoethnographical approach was used. Geographers Butz and Besio (2009) argue that authoethnography enables authors to become part of what they are studying, where the research subject becomes re-imagined via ‘reflexive narrators of self’. Autoethnography as method is becoming increasingly applied in human geography (Butz, 2010; DeLyser, 2015). Scarles and Sanderson (2016, p. 261) note in their work on tourism studies, that ‘…in autoethnography, subjectivity becomes constructive rather than destructive; accessing ‘hidden’ spaces, stimulating creativity and deepening connection … therefore, the researcher is becoming researched and this process can ultimately enable a far richer research engagement and insight.’ A number of health geographers have used autoethnography to capture the experience of place within the context of therapeutic landscapes. For example, Thompson (2021) employs autoethnography to reflect on personal encounters with digital health, and, in so doing, illustrates how digital health disrupts existing, and creates new, therapeutic landscapes. Liggins et al. (2013) use autoethnography to reconsider the inpatient unit as a place of healing and, in so doing, attend to not only the material world but the world within. Both these papers encourage the further use of autoethnography in therapeutic landscape inquiry.

The autoethnographic approach (Adams et al., 2022; Chang, 2008) used in this study not only allowed reflection on the experience of immersing within the three selected study sites, but provided the opportunity to observe others experiencing the sites of concern. When selecting the study sites, it was considered that the sites reflect different geographical landscapes in terms of nature and accessibility. Thus, the southern Westfjords reflects a rural cultural landscape located outside the popular ring road, the Jökulsárlón glacier lagoon reflects a popular tourist destination located by the ring road on the southeast coast, and the Icelandic Central Highlands reflects a wilderness area with limited access, especially during the winter months. Visiting in the fall, specifically in the month of October, these three sites were targeted in the following order: the southern Westfjords (3 days), the Jökulsárlón glacial lagoon (3 days), and the Central Highlands (3 days). All three sites were accessed by car or super jeep.

In keeping with autoethnographic method, the ‘I’ pronoun will be used from hereon in to describe the first author’s experience of the Icelandic landscape as a visitor. Data collection was similar across each site and included observation and documentation of the landscape and the associated activities and feelings experienced, which was facilitated through copious field notes, poetry writing, picture taking, and videorecording. This was complimented by observing and documenting the various activities that others at each of the sites concerned were engaged in. Therapeutic mobilities were limited to driving, walking, hiking, and boating. At times during this project I did feel like an interloper, as I was not only a visitor but also a researcher. Autoethnographic data captured the lived experience of these places through observing, hiking and being fully immersed in these sites. I used the power of observation in each of these sites, being cognisant of all senses, and the thoughts and feelings that were experienced.

The primary field data were my field notes and poems, which were entered into my electronic field book multiple times each day; the other data types supported these field notes. Analysis of data followed thematic analysis, which systematically organized and identified data into meaningful themes (Braun & Clarke, 2012). Thematic analysis procedures included creating preliminary codes, which were assigned to the data in order to describe the content. Themes were organized across the data from these preliminary codes. The themes were then reviewed, defined and named (Braun & Clarke, 2012). Following qualitative guidelines for assuring reliability and validity, research findings came to be transferable, dependable, credible and confirmable. In totality the natural, built, social and symbolic environments worked in synchronicity to produce three themes. The thematic results are presented using descriptive realist narrative and imaginative-creative poetry (Chang, 2008). Descriptive realist narrative portrays places and experiences as accurately as possible, while imaginative-creative writing allows the use of creative energy to express the autoethnographic experience in a range of genres, such fiction, poetry and drama (Chang, 2008). When combined, descriptive realist narrative and imaginative-creative writing create autobiographical poetry that depicts places and experiences in prose. Given the importance of situating the ethnographic researcher, the positionality and reflexivity of the first author is now briefly described.

I was introduced to the therapeutic landscape concept via Wil Gesler’s writings (1992) when studying as a doctoral student in social and health geography. Although not my central area of study, I became quickly captivated by the concept and engaged with the health geography community in developing it further. I published my first edited collection on therapeutic landscapes in 1999 (Williams, 1999); this laid the groundwork for a second collection published in 2007 (Williams, 2007). I was particularly interested in increasing engagement with the lesser-known spiritual aspects of therapeutic landscapes, engaging in pilgrimage sites both in real time (Williams, 2010) and virtually (Williams, 2013). I had the privilege of supervising numerous graduate students on the topic, ranging from green spaces for university student mental health (Windhorst & Williams, 2015a, 2015b), through to housing for both families with autistic children (Nagrib & Williams, 2017, 2018), and immigrant carer-employees working from home (Akbari & Williams, 2022).

A large part of my engagement with the therapeutic landscape concept was due to my interest in health geography, quality of life and well-being, as well as my own well-developed nature affiliation. My affiliation with nature began as a child, where I spent copious amounts of time outside, engaging in a wide range of natural spaces usually in an active way, whether gardening, cycling, swimming, cross-country skiing, playing a range of games, such as badminton, softball, golf, and hide-and-seek. My parents had the foresight of purchasing a cottage near Canada’s longest fresh-water beach when they started their family. Here, my siblings and I spent every summer and most weekends, as the majority of our out-of-school time was spent at the cottage, and much of it outdoors.

As one of my former graduate students revealed in his work with university student’s nature affiliation, positive experiences growing up in natural places have long-term mental health benefits given that they nurture connectedness with nature throughout the life trajectory (Windhorst & Williams, 2016). In this work, it was found that nature connectedness had a positive and significant correlation with students’ self-recalled positive childhood nature experiences, such as proximity to expansive, accessible natural places, and shared family engagement and valuing of these places.

My love of nature was nurtured further in high school, where I received summer employment as a junior conservationist. Spending time hiking in nature was a favorite pastime, and I jumped at the opportunity to go hiking elsewhere, whether outside my own community in the city or at my family’s cottage. In addition to hiking, long-distance cycling throughout North America was another therapeutic mobility that provided nature affiliation while in university. Having my own family provided the opportunity to raise my kids with many of these same activities, who now have high nature affiliation. As I move into my middle-age years, I am fortunate to have the opportunity to engage in work to conserve and protect natural places for future generations. This provides purpose and meaning while continuing to nurture my love of nature.

Having had the opportunity to visit Iceland in 2018, driving the famous ‘ring road’ around the island in search for coloured therapeutic landscapes, I was familiar with the white landscapes of the glaciers and ice, geothermal steam, and thousands of grazing sheep—all which contrasted with the black sand and basalt evident throughout the island (Brooke & Williams, 2021). The inconceivably peculiar but wild mountainscapes of Iceland quite consistently reflect this colour palette of white and dark colours. Going back to Iceland in 2021, I had the opportunity to spend an extended amount of time visiting what I had perceived in 2018 as three of the comparatively more wild and pristine areas of Iceland. Each of the selected sites will now be briefly described in turn.

Iceland’s Westfjords are often described as untapped nature, given that the famous ring road that circles the island does not include the many fjords making up the larger three fingers of the Westjords. Many would say that the lack of ring-road access has protected this part of Iceland, and particularly the northern tip of the Westfjords, which is primarily made up of the Hornstrandir Nature Reserve. This northern finger also contains Drangajökull, one of the many glaciers in Iceland. My visit was focused on the southern finger, and specifically the area around the largest populated village in this area, Patreksfjörður. An early snowstorm postponed the trip twice but, once there, provided a white snowscape to experience. I accompanied two Icelandic researchers on this 3-day excursion, both of whom were interested in land-use conflict between industry, tourism and other purposes. I too engaged in this work while visiting, as it provided a deep understanding of the villages we visited. We drove a rental vehicle with four-wheel drive in order to manage the snowy conditions of the roads. One of these researchers is a tourism geography researcher and co-author of this paper.

The second site, Jökulsárlón glacial lagoon became one of Iceland’s most popular tourist site in the years before the pandemic. Many travel books on Iceland describe the Jökulsárlón glacial lagoon as bewitching given the sense of awe it engenders amongst visitors. The glacial lagoon is located south of Vatnajökull, which is Europe's largest ice cap; its current size is approximately 18 square kilometers. It is attached to the Atlantic Ocean by a short waterway less than a kilometer in length. The lagoon has a very short history, both geologically and historically. It began to form around 1930 as a result of the retreat of the glaciers south of Vatnajökull ice cap, following the end of the Little Ice Age; the lagoon has been expanding rapidly since (Björnsson et al., 2001). Along with the growth in tourism in Iceland, the popularity of the site as a tourist destination has steadily increased. At this timeless site, the glacial water and glacial chunks flow into the Atlantic Ocean, leaving pieces of ice on the black coastal Atlantic beach. This beach, affectionately called Diamond Beach, is almost as popular as the lagoon. The lagoon shapes Breiðamerkursandur, the glacifluvial sand plain south and west of it. The glacial lagoon has characteristically cold blue glacial waters dotted with icebergs from the surrounding Breiðamerkurjökull glacier, an outlet glacier from the Vatnajökull ice cap. Seals visit the fish-filled lagoon. The lagoon is often described as one of the many natural wonders of Iceland not to be missed. I spent 3 days at this site on my own. While hiking along the lagoon up to the boundaries of the glacier, I spent hours observing the site at many different angles and altitudes. I took a Zodiac boat tour to get a fulsome understanding and experience of the expansive, deep lagoon, as well as the glacier tongue.

The third site visited, the Icelandic Central Highlands, was the most anticipated given that it is perceived to be the comparatively most wild. The Central Highlands are primarily located in the center of the country and make up a vast area of wilderness. As the country’s population are primarily found in settlements near the coast, with nearly 70% living in the Capital area (Statistics Iceland, 2022), the Highlands are primarily visited for outdoor recreation such as hiking, hunting, jeep touring, and cross-country ski touring. Part of the Highlands is accessible to all kind of cars in the summertime. In the wintertime, the area is less accessible, as there are no road maintenance services. Consequently, the Highlands are only accessible by super jeep in winter, given that they are equipped with 4 wheel-drive and have balloon tires that can be deflated for better traction in the snow. Grazing of animals, especially in the Highlands, has been greatly shortened in recent decades in order to protect the fragile vegetation; grazing is only allowed during the summer months. Iceland’s Central Highlands is often described as the largest span of wilderness in Europe. In years past, many in Iceland have tried to establish it as a national park and continue to advocate for its legislation. I was accompanied by an Icelandic super jeep driver who was very knowledgeable about the Highlands given that he had been a former member of Iceland’s Search and Rescue Team and, consequently, able to manage the worst weather and storm conditions.

## Thematic results

In keeping with the therapeutic landscape concept, the natural characteristics of the three sites studied were vibrantly clear, being augmented by built elements. The therapeutic social and symbolic characteristics of these environments were less apparent at first, only showing themselves as time was invested in experiencing the sites. Given the centrality of mountains as a key component of the landscape across all three sites, I was reminded and often felt their symbolic meaning throughout my visit. As with other mountainous regions across the world, Icelandic mountains not only symbolize strength, greatness and permanence, but also proximity to a heavenly existence and good health. Although perceived as claustrophobic by some, the general perception is that mountainous environments are healthful. A proponent of high-altitude medicine, Auer (1982) wrote that the stimulating powers of sun and snow in high altitudes exerts an influence on physical health, noting that a stay in high altitudes has been a long-standing medical prescription, remaining valid today with doctors recommending stays in high altitudes as good preventative medicine. He also summarized the spiritual rewards of an alpine environment: *‘He who knows how to open his mind and his heart in the mountains and to the mountains will be richly rewarded.’* (Auer, 1982, 18). There is no question that the mountains made me feel a sense of awe in their size and beauty, especially when painted with the various colours of white, grey, black, and green. Climbing mountains often symbolizes overcoming obstacles and making progress, as it did for me, described below as restoring my attention, reducing my stress and enhancing a more positive outlook.

The primary colours in the visited sites were white, black and green, although yellows, greys and browns were also evident in certain locales, providing bursts of colour. In totality, the natural, built, and symbolic environments worked in synchronicity to produce three thematic results: *restoration, awe* and *concern*. Overall, the thematic results, written below using descriptive realist narrative and imaginative-creative poetry (Chang, 2008), provided reduced stress, renewed attention, as well as enhanced physical, and psychological benefits for the researcher, as discussed in the first and dominant theme *Restoration*. *Awe* fed into feeling *restored*, while feeling more restored allowed me to experience greater *awe*. Feeling both *restoration* and *awe* engendered a sense of *concern* for the future of these natural landscapes.

### Restoration

Having been the glue that held my four-person nuclear family together safely and in good healthy throughout the first 1.5 years of the pandemic, I was ready for an adventure. The monotony of virtual teaching and working under lock down, coupled with the ongoing tasks of domestic management, and kids learning on-line due to stay at home orders took their toll on my wellbeing. Further, as a caregiver to my closest uncle, aged 88 years, who was living in long-term care and had experienced numerous outbreaks and subsequent lockdowns, I felt a great deal of worry and concern. As a university professor and parent, I was experiencing many of the symptoms of burnout characteristic of human service professionals: poor physical health, cynicism and negativity, all of which were reflected in a lack of energy and motivation, a change in sleep quality and appetite, and a loss of satisfaction from activities that previously were enjoyable (Kahill, 1988). In addition to needing a change and break from the everyday mundane activities of the pandemic, I was starving for wide open natural spaces.

In addition to feeling burnt out, I experienced a serious knee injury 5 weeks before travelling and had only 3 weeks of physiotherapy to heal and strengthen it. Luckily, I was able to purchase a knee brace for the trip. Throughout the trip I continued my regular daily exercises to ensure my knee was strong enough to endure the arduous hiking I was planning for. I did manage to complete all the hikes and walking tours I set out to do, but it was painful at times!

The limited hiking I did in the Westfjords and Central Highlands was primarily done with others accompanying me on these trips. These trips primarily include 1–2 h hikes, often on challenging terrain; having my hiking poles close by at all times assisted both my balance and confidence! As a consequence of having company on these hikes, there was a strong social component. Conversation often focused on the geological history, culture, or the people that characterized the place in which we were hiking or, in the case of the Westfjords, the research we were involved in there specific to the forces of globalization causing land-use conflict between industry and tourism. As a result of this project, which included collecting data via stakeholder interviews and focus groups, I was fortunate to speak to a number of Icelanders, both native and newly settled; consequently, I was able to more fully understand the forces at play that made the place work. I felt fortunate to be part of the research project as it allowed me to gather an ‘insiders’ understanding of the community and brought to life the physical geography of the fjords and surrounding geography. The Central Highlands experience also contained a strong social component, in that I was accompanied by a native Icelander who had a great depth of knowledge specific to the geography, culture and history of Iceland. He was generous in sharing his knowledge, which was often shared in the form of stories. He also knew the Highlands intimately, sharing the special places rarely written in tourist guides. We did quite a few hikes together, and he provided two opportunities to do two shorter solo hikes. Similar to the hiking in the Westfjords, conversation focused on the geography, culture and history of the places we were visiting. The solo hikes felt comfortable and safe given the safety net of knowing he was waiting at the end point. Although the social element in both the Westfjords and Central Highlands provided a more intimate understanding of both places, and even though I am still in touch with the folks who I travelled with, I still felt very much like a visitor, an outsider looking in.

The hiking I did around the Jökulsárlón glacial lagoon spanned both east (2 h) and west (4 h) of the main artery leading to the Atlantic Ocean. The eastern hike was shorter, following the lagoon’s beach. The western hike was more variable, crossing flood plains and hills. The western hike reminded me of the week-long tour de Mont Blanc that I did in the European alps as a university student – the scenic views of the mountains were ever changing. As I was alone in this site, I felt a strong sense of adventure, but also vulnerable given the vast and expansive landscape; into the second day of my stay I was yearning for social connection. What follows is a poem I wrote, describing the vulnerability of being alone and injured on these hikes:

*Trails of the Jökulsárlón Lagoon*

*So many trails to choose from,*

*Will my body hold up?*

*Climbing and climbing,*

*Knees and hips jarring with every step.*

*Can I do it?*

*Am I fit enough?*

*Where is my balance?*

*Use the hiking poles.*

*Are my hiking boots sturdy enough?*

*Pace yourself*

*This is not a race.*

Both hikes gave me a sense of the size of the lagoon, as well as the ecological processes at work. On both hikes I experienced the loud sound of glacier pieces calving, breaking into smaller pieces and hitting the water. On each occasion, a loud crack was heard, followed by the sound of the waves produced by the smaller pieces falling into the water. These calving processes are unplanned, as the glacier pieces calve at all hours and times of the day; I felt lucky to have experienced this, being present to and observant of the natural ecological processes at work. Otherwise, the soundscape is dependent on where you are located in the lagoon; there is the either silence accompanied by birds singing, or the gurgling of water under and around the glacier pieces. The cold arctic wind blowing down from the highlands was constant, being extremely strong at times and literally blowing me forward or backward on the trail, depending on which way I was travelling. I stopped to rest about every 20 min or so, finding a rock to sit on while observing the landscape. Here I listened, watched, and felt. Each time I was struck with the beauty and serenity of the experience. Artic terns flew above me, while water streamed out of rocks, flowing into the lagoon. The glacial ice floating in the lagoon melted before my eyes, provided a sense of timelessness, like I was in a backwards time warp. The experience of watching the ice melt as it floated toward the Atlantic Ocean felt like a sped-up movie reel; century old ice melting in a matter of minutes. Therapeutic mobilities were limited to walking/hiking, and boating in the lagoon.

Both the Westfjords and Central Highlands, with their majestic wild scenery, extent, and wide-open spaces, provided not only relief from stress but renewed attention, reflected in the goal setting I did in both these environments. Given the rugged terrain in all three places, hiking provided enhanced physical benefits while nature provided psychological benefits.

### Awe

Similar to the work of Pearce et al. (2017) who explored tourists views of a natural part of Tasmania, Australia, a range of awe-inspiring experiences were felt when visiting the three chosen sites, including: (1) vast geological landscapes (i.e., mountains and glaciers), (2) aesthetics (i.e., sculpted mountains, fjords, and glaciers of various blues/whites and transparencies) and fauna (i.e., sheep, seals and birds), (3) ecological phenomena (i.e., movement of tree line to higher altitudes, erosion of mountains, calving of glaciers, movement and flow of glaciers, tall waterfalls), and (4) reflective/perspective moments (i.e., mortality, timelessness). Each of these will be discussed in turn.

#### Vast geological landscapes

Iceland is made up of vast geological landscapes, which are characteristic of all the three sites of concern herein. The southern Westfjords have majestic mountains surrounding every fjord. The drive to and from the southern finger of the Westfjords was exceptionally scenic, as the road follows the coast of each uniquely beautiful fjord. Although the size of the mountainscapes made me feel miniscule, the beauty of each fjord made me feel and that I was in a perfectly wild place, with the greens, blues, whites and blacks working so beautifully in combination. I felt a great deal of gratitude for having experienced such beauty.

The Central Highlands are made of what appears to be a never-ending range of white, glacier-topped mountains with great extent and much folklore. In snow-covered Hveravellir, where we stopped for the first night, I received a tour of the many hot springs and spent some time in the geothermal pool. Here, the awesome story of the legendary Fjalla Eyvindur, which translates as “Eyvindur of the Mountains” (Iceland magazine, 2015) was shared. Eyvindur is the most well-known Icelandic outlaw in history, being the source of numerous myths and stories. Eyvindur fled into the highlands in the mid-1700s, being accused of stealing. He lived there for 20 years, evading the state that were consistently on the hunt for his whereabouts. The Westfjörds also has folklore about him, as Fjalla-Eyvindur lived there for a few years. These stories are taught in Icelandic elementary schools, as the tail of the outlaw is a story of resilience, determination, strength of body, spirit, and mind. How he could live for so long in such a vast, sparse and wild environment was bewildering to imagine.

The Jökulsárlón glacial lagoon is known as the jewel of Iceland for the tourism industry, having a backdrop of the massive white Vatnajökull glacier and the white and grey mountain ranges making up the Vatnajökull National Park. As with the mountains in the Central Highlands, Westfjörds, and in all parts of the world, there is a feeling of majesty and magnificence when looking at them. The mountain ranges and glaciers contribute to the aesthetics of these natural landscapes, as do the waterscapes, skies, and landform shapes; all reflect the nature-based colours of blue, white, green, brown, red, grey, and black. I felt beauty surrounding me in each of these sites, often having trouble deciding in which direction to look.

#### Aesthetics & fauna

The large spaces, extent, and uninterrupted spectacular natural views made me feel remarkably small and insignificant. The many fjords that make up the southern Westfjords were an icy blue, surrounded by steep mountains of grey, red and black – all of which were spotted with cascading rivers and green meadows. Sea birds were abundant in the fjords, making it an international birding destination. The many hues of blue and white within the Jökulsárlón glacial lagoon provided great contrast to the grey and black seals sunning themselves on the glacial ice. The large white glaciers atop the grey and black mountains in the Central Highlands were stunning given the vast brown and black rock deserts that stretched out on either side of the single-lane two-direction dirt road. Such multicoloured natural wilderness gave me a strong feeling of being alive, reflected in a experiencing a sense of excitement, adventure and hope for my life’s future.

#### Ecological phenomenon

A wide range of ecological phenomena, most related to climate change and glacial melt, take place in each of the sites of concern, but the visual effect is most rapid in the Jökulsárlón glacial lagoon. As referred to earlier, the lagoon experiences the calving of glaciers, as well as the movement and melting of the resulting icebergs out to the Atlantic Ocean. Various wildlife habitats are also evident, including a range of birdlife, fish, and harbor seals, the latter which congregate near the mouth of the lagoon to catch fish. One of the most fascinating processes is the melting of the many 1000-year-old glacial ice melting into the Atlantic Ocean within a 6-month window. The lagoon is perpetually growing, being formed naturally from melted glacial water; big blocks of ice calve off the ever-shrinking glacier. The rapidity of this ecological process is not only awe inspiring, but astonishing. The lagoon grows approximately 300 m each year, as the glacier’s tongue recedes, with a greater degree of calving and a greater volume of meltwater.

#### Reflective/perspective moments

The reflective moments in each of the natural sites were many. The Westfjords provided the first geothermal bath of the trip; located high in the mountains, it was a sight to watch the sky turn from blue to orange, then pink as the sun set over the fjord. Geothermal baths, recognized by others as socially and culturally responsive therapeutic landscapes (McIntosh et al., 2021), were a common occurrence in the Central Highlands as well, given the abundance of geothermal activity all over the island**.** Time in a natural geothermal bath provided time to reflect on the day, while providing restoration to sore muscles, and a full but tired mind. The timelessness of the Jökulsárlón glacial lagoon was where I simultaneously felt my mortality and brevity of life, together with gratitude for the beauty of the place. The expansiveness of the Central Highlands, with its many glacier-topped mountains and miles of moraine desert had great extent, provided perspective on the importance of sustainable development. As beautiful as the land was, it was the sky that seemed to come alive given its ever-changing nature, whether in the many hues of blue, white, orange and pink. The following poem was written about the sky in the Central Highlands:

*Highland Sky*

*Constant change, like a symphony of sound*

*Rain or snow falls*

*Clouds move in and over mountain tops*

*Mist falls in waves, wetting my face*

*The sun peaks out to join the orchestra*

*Playing hide and seek with the other elements*

*Before succumbing to mastering the sky*

*A rainbow presents itself as the climax of the concerto*

*Together with a backdrop of blue, grey and white*

*Were these the same skies of Eyvindur of the Mountains?*

### Concern

Concern was felt in all three sites, due to the effects of overtourism (Dodds and Butler, 2019), climate change impacts, and the Anthropocene. This was evident and symbolized by: the litter at the Jökulsárlón glacial lagoon; land conflicts with tourism in the Westfjords, and; a growing network of roads in the Icelandic Central Highlands. The expanding road network in the Highlands unlocks these wild landscapes for infrastructure development and resource exploitation (Sæþórsdóttir & Ólafsdóttir, 2017; Tverijonaite et al., 2018), primarily in the areas of tourism and energy harnessing (Tverijonaite et al., 2019). All three sites, and particularly the glacial lagoon and Central Highlands, showed clear signs of being impacted by climate change. As with elsewhere in the world, the Icelandic glaciers are melting at an alarming rate, having clear impacts on nature-based tourism, especially in the southeast of the country (Welling et al., 2020). The Breiðamerkurjökull glacier started to retreat due to rising temperatures, and between 1930 and 1940 the first signs of what now is known as Jökulsárlón glacier lagoon became evident. The lagoon has been growing in size ever since, due to a warming climate. Rising temperatures continue to shape the lagoon, which is currently the deepest lake in Iceland and growing four times larger since the 1970s (Björnsson et al., 2001; Guðmundsson et al., 2017). In addition to receding glaciers, the warming climate is evident in the slow creep of the tree line, evident in the new growth of native birch trees, albeit stunted.

Tourism ballooned in Iceland at the turn of the century, following the country’s economic collapse. The central government initiated a highly successful marketing campaign to boost tourism in Iceland. Consequently, many of the most popular hiking trails are over trodden. This is, for example, the case for the long-distance hiking trail between Landmannalaugar and Þórsmörk in the Southern part of the Central Highlands of Iceland. To illustrate the rapidity of the growth of the tourism sector, the second author recalls setting this trail with direction markers back in the 1980s and, in 2021, the trail is recognized as one of the most popular in Europe. Over trodden trails are not the only concern, as congestion of both people, vehicles, and buses at the Jökulsárlón glacial lagoon and Diamond Beach has initiated the building of two additional paved parking lots on the Diamond Beach side of the Ring Road.

Tourism people and people bring pollution. Both in the Jökulsárlón glacial lagoon and in the Central Highlands, I found myself picking up garbage, and specifically plastic bottles, bags and rope of various kinds. Tourism has brought garbage, and plastic specifically, over what was formerly known as pristine wilderness. Many tourists appeared to be visiting the Jökulsárlón glacial lagoon to check the site off their list of places to see while visiting Iceland. This idea of a tourist site checklist was brought up in discussion with community stakeholders in the Westfjords as well. I felt concern and worry over the changes that tourism has brought to these natural environments, in the form of building, road construction, sign pollution, and all other forms of pollution – garbage, sound, and plastic.

The developing road network in the Central Highlands is a sign of potential infrastructure development. Visiting one of the oldest and most remote hiking huts in the Central Highlands (constructed in the 1940s) demonstrated the very basic accommodations once available – bunk beds and an outhouse. Further up the main dirt road is Hveravellir, a reconstructed hiking hut located beside a natural hot spring area. The newer hotel beside it is equipped with intranet, flush toilets, hot water showers, and sophisticated kitchen facilities, all of which have become available year-round since a power and intranet line was dug across miles and miles of the Central Highlands a few years ago. Road access allows such infrastructure to be built, providing the foundation for further development. Although there are many protected areas within the Central Highlands, the whole area is not currently protected. The increased interest in experiencing this last bastion of wilderness in Europe continues to grow. This is evident in the large busses, equipped with all-wheel drive and balloon tires, taking tourists into the Highlands for day trips through to winter, when accessibility becomes more limited. I felt concern over the need to protect the whole area, given the growing popularity of the Central Highlands as a nature-based tourist destination. Driving along the snowy road toward Hveravellir we rescued two tourists whose vehicle had gotten stuck in the snow; they had been stranded for some time. Although they had rented a 4-wheel drive vehicle, they wrongly assumed they were able to transverse the highlands. Once out of the snow, it was suggested they head down to the coast and to not veer from the Ring Road. According to my driver/guide, such an incident is commonplace in the Highlands, happening far too often.

Sheep farming is one of the largest agricultural sectors in Iceland, with thousands of sheep freely roaming during the summer months in most areas outside of the capital area of Reykavik. Given that rural areas make up 90% of the island, there is much opportunity for sheep to freely roam. Sheep are very much a part of the natural landscape, whether in farmyards, fenced off fields, or grazing near the roadside or up in the Highlands. Gorman (2017) discusses the need to incorporate animals in the study and understanding of therapeutic landscapes, suggesting the need for them to be understood as co-constituents and co-participants of therapeutic spaces. Using examples of how animals are agents in the therapeutic encounter for children with learning challenges, such as dyslexia and ADHD, Gorman highlights the needs to ‘bring the animals back in’ (p. 329). Certainly, the sheep give life to the landscape, not only with their movement but with the pleasant ‘singing’ sounds they make. Hiking past the yews and lambs was a real treat, allowing close proximity and observation of the beautiful animals. Although there are black sheep amongst the many white sheep, most of the Icelandic sheep are white. Farmers keep the yew and lambs together on the farm early in the spring. Once the lambs are established and firmly bonded with the yew, the sheep are let out to freely roam the pasture all summer long, colouring the landscape with various intensities of white, depending on the number. They are collected in the fall using dogs, horses, and various mechanized vehicles. Sheep were still roaming free when I visited the Westfjords in October, when I wrote the following poem:

*Pristine Wilderness in the Westfjords for How Long?*

*Miles beyond any village, town or hamlet*

*Lays a magical spot,*

*Where waterfalls flow.*

*Towered by heighty, wind-swept mountains of various shapes,*

*The healing colors of blue and green are balanced*

*By the grey mountain stone and black lava rock.*

*Interested sheep look straight through me.*

*No trails, no signs, no name*

*Just pure beauty in its finest – for now.*

*Worry about global forces invades my mind,*

*Globalization, climate change, population growth and overtourism.*

*How can we best manage?*

Although all three sites had unique therapeutic characteristics which contributed to healing burnout, the Westfjords were comparatively most healing, followed by the Central Highlands and the lagoon. This order was primarily due to the influence of the extent of the social component in the first two most healing sites, complimenting the natural and symbolic components.

The results of this study speak to one of the most immediate discussion points, that being the need to act to encourage sustainable tourism development, especially in the Sub-Arctic and Arctic where changes due to climate warming are happening at a faster rate than elsewhere. The concern over how natural environments are being impacted by climate change motivates action, whether via micro or macro-scale changes. This certainly occurred for me with respect to my everyday life post-travel, as I consequently swapped my clothes drier with a drying rack, purchased an electric car, and accepted an invitation to join the Board of Directors for a community food garden that I volunteer for in the summer. This transformative impact provides promise that the same will be experienced by the thousands of millions of travelers seeking nature-based tourism, further building awareness of our environmental impact and building agency around sustainability, and sustainable living more broadly.

## Discussion

Using an autoethnographic approach to the experience of Icelandic natural areas through the lens of therapeutic landscapes provides four discussion points. First, as with earlier work on wilderness as therapeutic landscape (Williams, 1999; Windhorst et al., 2015a, 2015b, 2016; Bell et al., 2018), the first thematic finding provides further evidence that nature provides cognitive restoration. The autoethnographic researcher’s experience of the Icelandic natural landscape via the three study sites was restorative. This feeling of being restored was evident when returning back home feeling refreshed, re-energized, and ready to engage in both academic work and family/home management/care work once again. Given that therapeutic landscapes theory understands place as a key element in engendering health and wellbeing, through physical (natural and built), social, and symbolic environments (Bell et al., 2018; Gesler, 1992), the three sites of concern herein highlight the healing power of the natural environment. Employing autoethnography, this paper has emphasized the experiential, embodied and emotional geographies from the researcher’s perspective via three thematic findings. *Awe* feeds into feeling *restored*, while feeling more restored allowed one to experience greater *awe*. Feeling both *restoration* and *awe* engendered a sense of *concern* for the future of these natural landscapes.

Related to this is the variable of coloured landscapes, or ‘palettes of place’ (Bell et al., 2018). The healing blues and greens were found across all three sites, complimented by the awesome yellows, browns, and greys. Given the presence of water, recognized by environmental psychologists (Kaplan, 1995; Kaplan & Kaplan, 1989) and health geographers (Foley, et al., 2019) as the most healing natural component of landscape, the various colours of water in Iceland – whether blue, grey, bubbling white, or mineral rich green, was apparent across the three sites. Blue was the dominant colour of the ocean surrounding the many fjords in the northwest, and the coastline surrounding the lagoon in the south. Building on earlier work (Brooke & Williams, 2021), white was also dominant, given the vast amount of moving water, whether found in the many rivers and falls of the Westfjords, Central Highlands, or lagoon site. Mineral-rich green water was evident in the Central Highlands and the Westfjords, specifically in areas rich in geothermal baths and geysers. In addition to white water, white landscapes dominated each of the sites given the presence of thousands of sheep, and ice and snow via the substantial, numerous glaciers. The colour white often contrasts with other colours, such as: the black sand on Diamond Beach adjacent to the glacier lagoon, the green fields and pastures of the Westfjords, and the grey rock and brown soil of the Central Highlands.

A third discussion point addresses the possible exclusionary attributes of these sites (Bell et al., 2018). With respect to the exclusionary nature of the identified sites of concern, two points are most evident – cost and accessibility. Costs of travel in Iceland, like most of Europe, is expected to exclude many potential visitors; those who visit and travel in Iceland are generally well-off given the high costs of services when compared to many other equatorial tourist destinations. Surprisingly, access to the three sites of concern herein, as with most natural places in Iceland, is free, still having no entrance fee in place. Free access seems, however, to be slowly changing as a parking fee is now in place in a few popular tourist destinations, such as in Vatnajökull national park, and in Þingvellir national park. Increasing, a number of other sites, especially private ones, are requiring an admission fee and/or payment for using the restrooms (Øian et al., 2018). Increased tourism in Iceland has furthermore changed accessibility demands, both with respect to volume of access to all popular tourist destinations, and with increased accessibility for those in wheelchairs and/or who use mobility aids. One development noted by the autoethnographic researcher, since the earlier 2018 trip to the glacial lagoon, was the addition of four parking spots allocated to those with disabilities. These spots were strategically placed to allow access to the waterway trail between the Atlantic Ocean and glacial lagoon. This improved accessibility brings nature closer to more people, allowing greater inclusivity and therefore a greater number of visitors to reap the positive health benefits that nature provides. However, many researchers have shown that increasing accessibility to a greater number of tourists leads to landscape change, transforming wilderness to developed and often populated areas and, subsequently, also changing visitors’ experience of it (Bishop et al., 2022; Haraldsson & Ólafsdóttir, 2018; Ólafsdóttir & Haraldsson, 2019; Tverijonaite et al., 2018). It is therefore a great challenge to manage nature-based tourism in wilderness settings like the Icelandic Central Highlands.

## Implications for nature-based tourism in a post-COVID era

This study has many implications for nature-based tourism given that therapeutic landscape theory intersects with cultural ecosystem services. There are many benefits that natural places provide, one of which is the contribution they make to enhanced mental and physical wellbeing (Williams, 1999; Windhorst et al., 2015a, 2015b, 2016; Bell et al., 2018); this benefit provides considerable opportunities for nature-based tourism in a post-COVID era. After 2 years of isolation and stay-at-home orders at various levels, COVID has opened our eyes to the therapeutic values of nature. As confirmed by the results of this study, research evidence notes that spending time outdoors in nature has been a critical factor enabling people to cope with the stress in and following the pandemic (i.e., Mental Health Foundation, 2021). Further, with the growing number and intensity of natural disasters brought on by climate change across the world, crisis such as the COVID pandemic has alerted us to how quickly our world can change. The experience of the pandemic amplified concern for climate change while increasing public support for a green recovery, suggesting bolder climate policies and greater interest in sustainability (Mohommad & Pugacheva, 2022).

As outlined by the United Nations Sustainability Development Goals (2021), sustainability is a key dimension in our planet’s wellbeing which, in turn, impacts society's wellbeing. Sustainable development has been a key theme in Iceland’s government tourism strategy for the past 30 years and, in so doing, acknowledges its importance for Icelandic tourism. In 2019, the Icelandic government set out an ambitious vision for Icelandic tourism through to 2030, where the goal was to become a leader in sustainable development worldwide (Government of Iceland, 2019). Although sustainable development is the goal, the economic dimension of sustainability has consistently been a leading force in Icelandic tourism, somewhat reflecting the laissez-faire approach largely guiding the tourism sector. Ólafsdóttir (2021) points out that with increased knowledge of sustainable development, there is greater acceptance that the three pillars of sustainability, defined as economy, society and nature, are part of a closed system; nature sets limits for societal growth, and society sets limits for economic growth. It is therefore necessary to understand the behavior of the system in order to know where the boundaries between these pillars are and, consequently, manage development so that it remains within a sustainable system. A holistic vision of all the influencing factors, and an understanding of how they interrelate, is therefore fundamental to the development of sustainable tourism.

As noted earlier, COVID has brought a time of pause for tourism, thus providing an opportunity to critically reconsider tourism challenges, such as overtourism and impacts of climate change (Gössling et al., 2021). Moreover, this pause has given precious time to better improve the planning and management of tourism in meeting the goal of the sustainable development for therapeutic nature-based well-being. For example, this time of respite allowed Iceland to renovate sites experiencing overtourism, such as the Jökulsárlón glacial lagoon. In addition to extending the number of parking spots, signed accessible parking spots have been allocated for disabled folks, making use of the space adjacent to the Jökulsárlón glacial lagoon on the Diamond Beach site.

In addition, achieving ecological sustainability, natural tourist destinations should involve interpretation, education, and enjoyment of nature, while bringing benefits to both visitors and local communities. Related to this is the issue of safety. In each of the three sites of concern, many of the features are viewed at the tourist’s risk, with limited signage, direction, or rules. Trained as a National Lifeguard in Canada, the autoethnographic researcher often caught themselves worrying about the lack of safeguards, such as fences and other such barriers, at the sites of concern. The limited safety protection translates into somewhat of a hazard for some, such as those: with young children, shaky on their feet, or overly zealous photographers. Exploring whether this was a concern for others would be a useful follow-up study. Hence, when developing nature-based tourism, it is critical to develop site-specific zoning for the different market groups, using focal points as a management tool (Ólafsdóttir et al., 2018). In this way, it is possible to protect the most pristine wilderness (Sæþórsdóttir & Ólafsdóttir, 2017).

Iceland’s nature-based tourism sites exist on a continuum, from minimally developed to fully commercialized. In the most remote and wild places, such as the Central Highlands, visitors must often rely on their own GPS navigation systems to find the many natural treasures. In the most popular Highland areas, there is a volunteer Search and Rescue Team stationed during the summer high season and, so far, they have been very quick to respond when they receive a call for help. Further, mobile phone connection is now available across a large part of the Highlands. Whether this means that the therapeutic value of the place improves (given a potentially enhanced feeling of safety) or declines (given that potential of sources of stress are much more proximal) is important to explore in a follow-up study. Although limited in number, Iceland has commercialized some of its natural sites. Near one of the entrances to the Central Highlands, two geological attractions are found: Gullfoss waterfall and Geysir geothermal area. In both these sites, we find a large food court, gift shop and multiple hotels. In fact, unlike many of the natural sites throughout the country, these natural sites have built paths, meant to steer the crowds. Although these commercialized sites are accessible to those with mobility aides, the autoethnographic researcher much preferred the sites which were not commercialized, as their natural quality and, consequently, their therapeutic value remained much more intact. Exploring the therapeutic benefits of limited development versus commercialization, as seen with mass tourism, provides yet another topic for further studying this realm.

## Conclusion

Framed within the therapeutic landscape concept, this autoethnographic study of three of Iceland’s natural sites adds to the understanding of the healing effects of the multi-colour natural landscapes of Iceland in a COVID era. In sum, the natural sites of the southern Westfjords, Jökulsárlón glacial lagoon, and the Central Highlands worked in synchronicity to produce three thematic results. The three themes of *restoration, awe*, and *concern* provided renewed attention, reduced stress, as well as enhanced physical and psychological benefits for the autoethnographer researcher. Further, this paper has highlighted how health and tourism geographers are well positioned to work collaboratively to sustain the therapeutic elements of natural landscapes, recognized as a cultural ecosystem service. In so doing, they can influence sustainable development and consumption through proper and appropriate planning and development of such tourism destinations.
